# Novel insights into causal associations of body mass index or height with pneumothorax: a two-sample Mendelian randomization study

**DOI:** 10.3389/fnut.2024.1391017

**Published:** 2024-07-22

**Authors:** Gengqiu Liu, Dongqing Yan, Xiaohuai Wang, Anbang Liu, Junhang Zhang

**Affiliations:** ^1^Department of Thoracic Surgery, The Seventh Affiliated Hospital, Sun Yat-sen University, Shenzhen, China; ^2^Department of Thoracic Surgery, Qingdao Municipal Hospital, Qingdao, Shandong, China

**Keywords:** body mass index, height, pneumothorax, Mendelian randomization, causal association

## Abstract

**Background:**

Observational studies have reported an association between body mass index (BMI) as well as height and the risk of pneumothorax. However, it has long been unclear whether BMI or height are causally associated with pneumothorax.

**Methods:**

Genetic summary data for BMI, height and pneumothorax were retrieved from multiple independent large genome-wide association studies (GWAS). A series of quality control steps were conducted to select instruments. Four independent two-sample Mendelian randomization (MR) analyzes were performed to adequately assess the causal relationship between BMI or height on pneumothorax, and the robustness of the results was assessed by a series of sensitivity analyzes.

**Results:**

Height increased the risk of pneumothorax with an OR of 1.5181 (95%CI 1.3092–1.7604; *p* = 3.28e-08); there was no evidence of a causal effect of BMI on the risk of pneumothorax with an OR of 0.8979 (95%CI 0.7417–1.0869; *p* = 0.269). Height increased the risk of spontaneous pneumothorax with an OR of 1.0010 (95%CI 1.0002–1.0018; *p* = 0.012); the results showed no significant causal relationship between BMI and spontaneous pneumothorax either with an OR of 0.9992 (95%CI 0.9983–1.0002; *p* = 0.112).

**Conclusion:**

Our results supported a genetic association between height and pneumothorax. We found that height increased the risk of pneumothorax. However, no evidence was found to suggest a causal relationship between BMI and pneumothorax risk. The relationship between BMI and pneumothorax requires further in-depth analysis.

## Introduction

Pneumothorax refers to the state caused by gas entering the pleural cavity, which is divided into spontaneous pneumothorax and traumatic pneumothorax (1). In the clinic, patients with spontaneous pneumothorax usually present with a sudden onset of chest pain with or without dyspnea and asymptomatic patients are sometimes found on a chest CT or X-ray (2). The causes and mechanisms of spontaneous pneumothorax are currently unknown, and a number of associated risk factors have been previously reported, including smoking, male sex, low body mass index (BMI), height, toxic metals, and environmental factors (3, 4). Notably, lower BMI and higher height have been identified as the most likely and common risk factors for triggering pneumothorax in several previous observational studies (2, 5). However, it is difficult to rule out the influence of confounding factors and reverse causation in observational studies and to convincingly establish a causal relationship between height or BMI and spontaneous pneumothorax. Randomized controlled clinical trials (RCTs) are also often difficult to conduct because of ethical concerns.

Mendelian randomization (MR) is an approach that uses single nucleotide polymorphisms (SNPs) as an instrumental variable (IV) of exposure factors to infer causality between exposure factors and outcomes, minimizing bias due to confounding factors and reverse causation as far as possible (6, 7). The strength of evidence from MR is lower than RCTs, but stronger than in observational studies such as case control studies (8). In addition, MR studies typically include much larger sample sizes than clinical trials, particularly when clinical prevalence is low.

According to our current understanding, no research has employed the MR Approach to examine the causal relationship between height or BMI and pneumothorax. Therefore, this study aimed to test whether exist a causal relationship between height or BMI and the risk of pneumothorax by two-sample MR Analysis.

## Methods

### Study design

**Figure 1:**
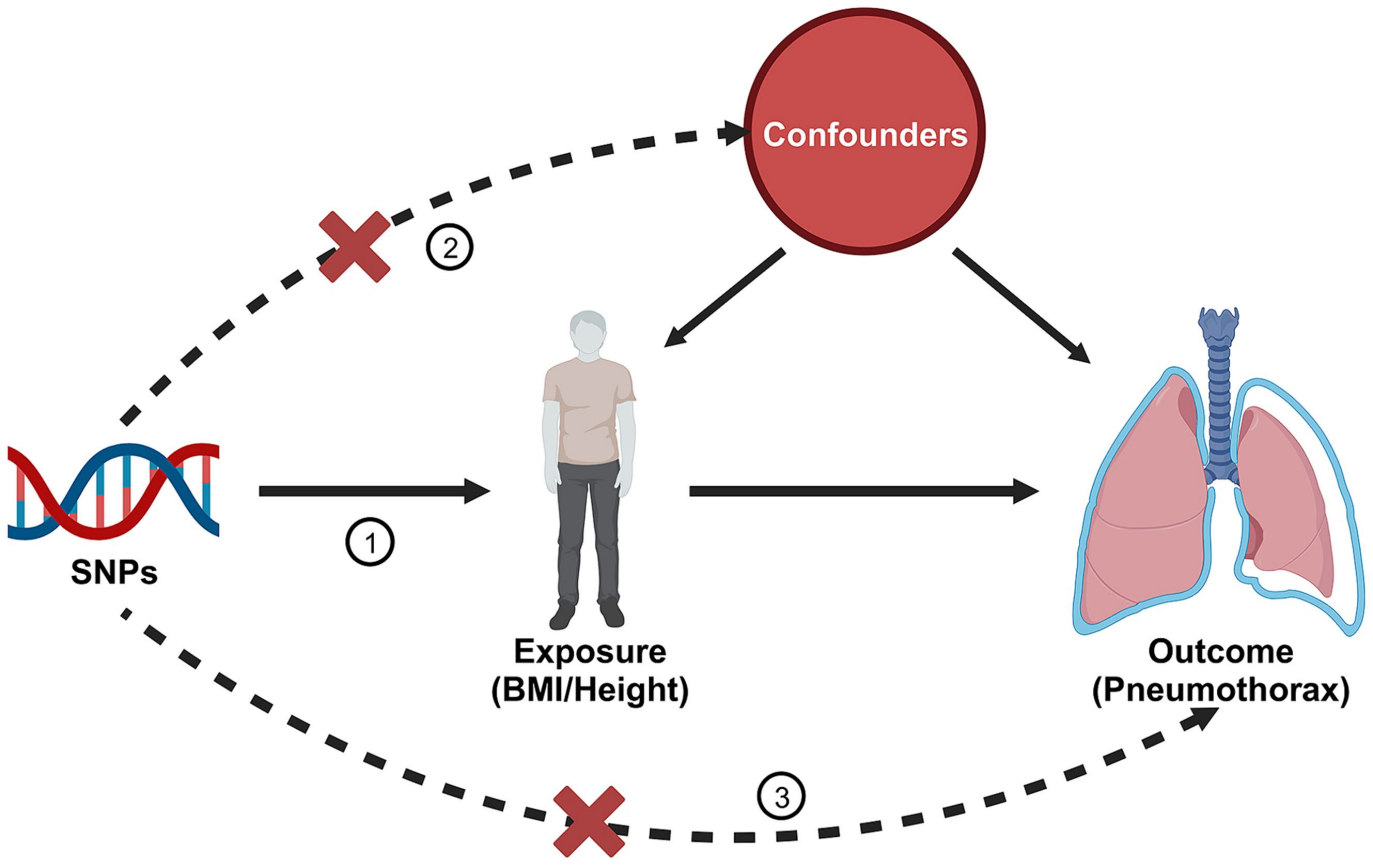
Three assumptions of Mendelian randomization. Created with BioRender.com.

To effectively demonstrate causal effects, SNPs used as IVs in MR analyzes must fulfill three key assumptions: (1) the assumption of relevance: the IV must be strongly associated with the exposure; (2) the assumption of independence: the IV must not be associated with any confounders related to the outcome; (3) the assumption of exclusionary restriction: the IV must influence the outcome only through the target exposure, and not through any other pathway to influence outcome (Figure 1) (9). All datasets included in this study are publicly available GWAS abstract data and ethical approvals were obtained in all original studies.

### Data sources

The BMI, height and pneumothorax data were obtained from a large cross-ethnic human genome-wide association study (10). This study integrated European human trait genome mapping data from the UK Biobank and FinnGen databases, and expanded on the deep phenotype genome-wide association study of East Asian populations from the BioBank Japan database. Most of the data are from populations of European origin. This study not only improved the resolution of the human trait genome map, but also provided a potential avenue for the re-investigation of human diseases through genetics. The SNPs associated with BMI and height were derived from 359,983 and 360,388 samples of European origin in this study, respectively. The SNPs associated with pneumothorax were derived from 1,747 pneumothorax cases and 475,987 controls of European ancestry provided in this study. Statistical summary data of spontaneous pneumothorax were obtained from the UK Biobank (11). This dataset provided 1,097 cases of spontaneous pneumothorax and 461,836 controls of European origin (12). More detailed information is available in the original article. Details of the data sources were provided in Supplementary Table S1.

### Selection of IVs

A series of quality control measures were implemented to incorporate suitable genetic IVs for subsequent MR studies (13). Initially, we isolated SNPs associated with BMI or height exhibiting genome-wide significance (*p* < 5 × 10^−8^) (14). Second, to maintain the independence of genetic variables, we executed the aggregation process utilizing the linkage disequilibrium estimations computed by the Europeans within the 1,000 Genomes Project (criterion for *R*^2^ < 0.001, window width = 10,000 kb) (15). Third, we eliminated SNPs linked to potential confounders, such as smoking, age and gender and the traits related to each SNP can be explored at Phenoscanner^1^ (16). Subsequently, we extracted the GWAS summary data for the chosen SNPs from the result dataset and eliminated those SNPs that demonstrated a significant association with the outcome (*p* < 5 × 10^−5^). In addition, we harmonized exposure and outcome SNPs to maintain consistency of effector alleles and removed palindromic SNPs and that were not present in the outcome. In final, we conducted MR-PRESSO (Pleiotropy RESidual Sum and Outlier) global tests to pinpoint disparities among genetic connections established by various genetic variations and to eradicate outlier genetic variants (17).

In order to mitigate the risk of potential weak IV bias, we derived the F-statistic employing the following formula: F = *R*^2^(*n* – k–1)/k (1–R^2^). Here, *R*^2^, *n*, and *k* refer to the proportion of the exposure variation accounted for by the chosen genetic instruments, the sample size of the exposure GWAS, and the number of selected genetic instruments, respectively. SNPs with a F-statistic ≥10 indicate that the selected SNP is valid and sufficient strength (18). Therefore, SNPs with a *F* < 10 were excluded from the MR analysis.

### Mendelian randomization analysis

In this research, we conducted four independent two-sample MR analyzes. To avoid potential pleiotropy effects of genetic variation, we used four different MR analysis methods to assess the causal effect of BMI or height on pneumothorax. The primary MR analysis was executed utilizing the inverse variance weighted (IVW) method, regarded as the most robust (19). However, this method necessitates the absence of pleiotropy, meaning that the selected genetic variants employed as exposure instruments cannot influence the outcome through other pathways. In addition, we applied three other analytical methods (MR-Egger, weighted mode, weighted median) Moreover, we implemented three additional analytical techniques (MR-Egger, weighted method, and weighted median) in the present research to validate the reliability of our results. The application of the MR-Egger method generated reliable estimations, taking into account pleiotropy and assuming the invalidity of all instruments, albeit with minimal statistical power (20). The weighted median method produces unbiased causal estimates provided that at least half of the weights arise from valid instrumental variable (21). We also conducted leave-one-out analyzes to ensure that the effect of any single SNP did not distort the resulting causal relationship. In all our analyzes, *p* < 0.05 was considered statistically significant.

### Sensitivity analysis

We conducted sensitivity analysis to identify any presence of horizontal pleiotropy, which goes against the primary MR assumptions. Thus, we conducted the Cochran *Q-*test, MR-Egger intercept test, MR-PRESSO, MR Steiger test, leave-one-out analysis, and funnel plots to ascertain the presence of heterogeneity and horizontal pleiotropy to guarantee the reliability of our results. In particular, the Cochran *Q*-test was utilized to evaluate heterogeneity, with no notable heterogeneity observed if the *p*-value surpassed 0.05. The evaluation of horizontal pleiotropy involved calculating the intercept term derived from MR-Egger regression, and potential bias was indicated if this intercept term did not match 0 (20). The possibility of reverse causality between exposure and outcome was investigated using the MR Steiger test (22). Leave-one-out analyzes were conducted to check for any pleiotropy caused by individual SNP.

All MR analyzes in this study were conducted utilizing the TwoSampleMR software package (version 0.5.6) within the R programming language (version 4.2.2) (15).

## Results

### IVs screening

After a series of quality control selection and harmonization, we obtained multiple SNPs in BMI and height GWAS for MR analysis, respectively. The F statistic values of all IVs were > 10. All SNPs utilized in the four independent MR analyzes were detailed in Supplementary Tables S2–S5.

### Relationship between BMI and pneumothorax

**Figure 2:**
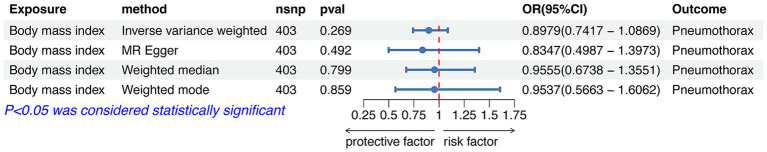
Forest plot of the associations between BMI with the risk of pneumothorax. CI, confidence interval; OR, odds ratio; SNP, single nucleotide polymorphism.

IVW analysis showed no significant association between BMI and risk of pneumothorax (OR = 0.8979, 95%CI 0.7417–1.0869; *p* = 0.269). The results of the remaining three analyzes showed no significant association between BMI and the risk of pneumothorax (all *p* > 0.05) (Figure 2). The above results implied that there was no causal relationship between BMI and the risk of pneumothorax.

### Relationship between height and pneumothorax

**Figure 3:**
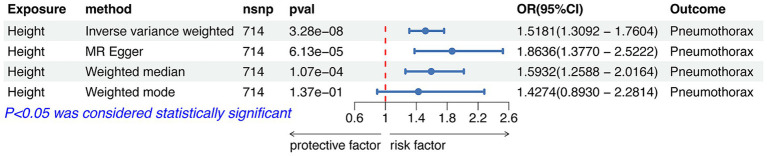
Forest plot of the associations between height with the risk of pneumothorax. CI, confidence interval; OR, odds ratio; SNP, single nucleotide polymorphism.

**Figure 4:**
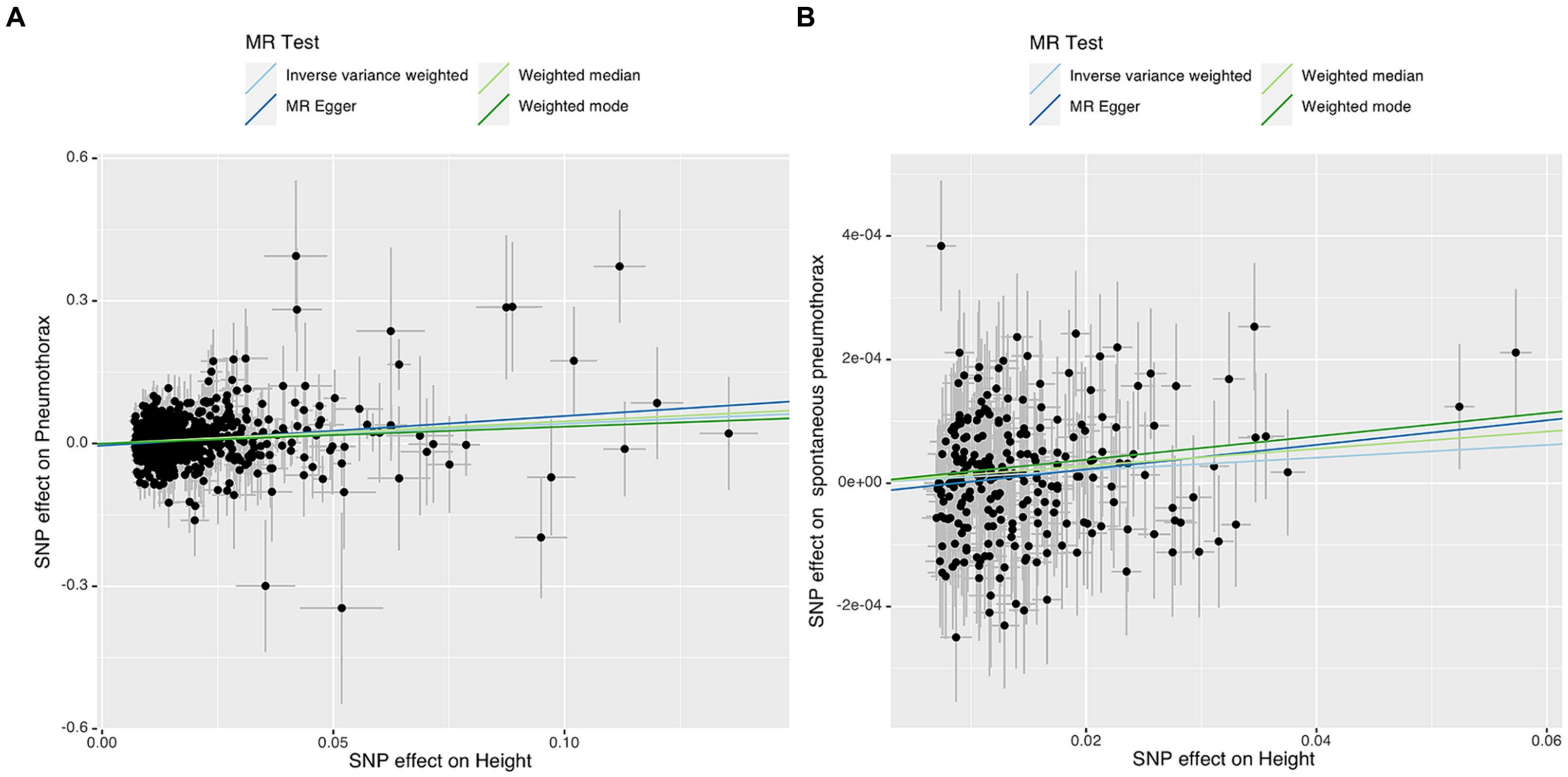
Scatter plot of the associations of genetic variants with height and the risk of pneumothorax **(A)** or spontaneous pneumothorax **(B)**. MR, Mendelian randomization; SNP, single nucleotide polymorphism.

IVW analysis showed that height was causally associated with the risk of pneumothorax (OR = 1.5181, 95%CI 1.3092–1.7604; *p* = 3.28e-08). Results of Weight median and MR-Egger showed significant and consistent direction. The results of Weight mode and MR-Egger were not significant but the OR had consistent direction (Figure 3). The above results indicated that the risk of pneumothorax also increases with higher height, and this trend was also demonstrated in the scatter plot (Figure 4A).

### Relationship between BMI and spontaneous pneumothorax

**Figure 5:**
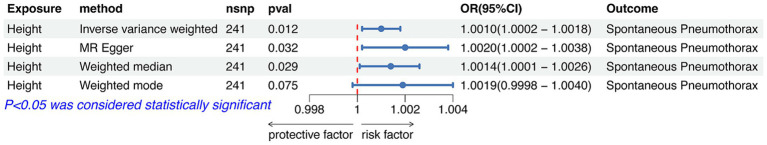
Forest plot of the associations between BMI with the risk of spontaneous pneumothorax. CI, confidence interval; OR, odds ratio; SNP, single nucleotide polymorphism.

After rigorous screening, we finally obtained 170 SNPs for analyzing the causal relationship between BMI and spontaneous pneumothorax. IVW analysis showed no significant association between BMI and the risk of spontaneous pneumothorax (OR = 0.9992, 95%CI 0.9983–1.0002; *p* = 0.112). The results of the remaining three analyzes showed no significant association between BMI and the risk of spontaneous pneumothorax (all *p* > 0.05). Detailed MR analysis results were presented in Figure 5. The above results indicated no causal association between BMI and risk of spontaneous pneumothorax.

### Relationship between height and spontaneous pneumothorax

After rigorous screening, we finally obtained 241 SNPs for analyzing the causal relationship between height and spontaneous pneumothorax.

**Figure 6:**
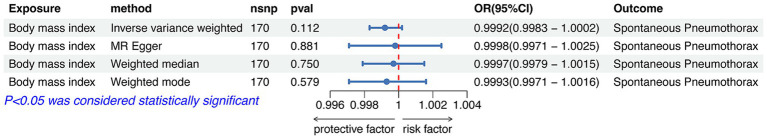
Forest plot of the associations between height with the risk of spontaneous pneumothorax. CI, confidence interval; OR, odds ratio; SNP, single nucleotide polymorphism.

IVW analysis showed that height was causally associated with the risk of spontaneous pneumothorax (OR = 1.0010 95%CI 1.0002–1.0018; *p* = 0.012). The results of Weight median and MR-Egger showed significant and consistent direction. The result of Weight mode was not significant but the OR had consistent direction (Figure 6). The above results indicate that the risk of spontaneous pneumothorax also increases with higher height (Figure 4B).

### Sensitivity analyzes

We have extensively validated the robustness of the results from four independent MR analyzes through a comprehensive range of sensitivity tests, including Cochran’s *Q-*test, MR-PRESSO global test, MR-Egger intercept test, and MR Steiger test (Table 1). In both MR-PRESSO global test and MR-Egger intercept test, all *p*-values were > 0.05, indicating that there was no horizontal pleiotropy throughout the analyzes. The MR Steiger test failed to identify any evidence of reversed causality, thereby corroborating the reliability of the established causal direction. All *p*-values obtained from the analysis of Cochran’s *Q*-test were > 0.05, suggesting the absence of heterogeneity. The other analyzes did not reveal any heterogeneity. Furthermore, the risk estimates remained relatively stable even when one SNP was excluded each time during the leave-one-out analysis. This suggested that no particular SNP played a crucial role in the causal association (Supplementary Table S6–S9). In addition, the funnel plots demonstrate a symmetrical change in effect size around the point estimate, suggesting that there is no significant evidence of horizontal pleiotropy (Supplementary Figure S1).

**Table 1:** Sensitivity analysis of four independent two-sample MR analyzes.

Exposure	Outcome	Cochran *Q-*test	MR-PRESSO	MR-Egger		MR Steiger test direction
		*p*-value	*p*-value	Intercept	*p*-value	
BMI	Pneumothorax	0.51	0.51	1.38E-03	0.77	True
Height	Pneumothorax	0.13	0.13	−4.29E-03	0.13	True
BMI	Spontaneous pneumothorax	0.63	0.64	−9.91E-06	0.66	True
Height	Spontaneous pneumothorax	0.85	0.85	−1.74E-05	0.25	True

BMI, body mass index; MR-PRESSO, Mendelian randomization pleiotropy residual sum and outlier.

## Discussion

Pneumothorax is a common and urgent clinical problem worldwide (23, 24). Treatment options for pneumothorax depend on the severity of the pneumothorax, the overall health of the patient, and the cause of the pneumothorax (25). Although there have been significant advances in the treatment of pneumothorax in recent years, particularly in surgical techniques and conservative management (26–28), we still recommend that prevention of pneumothorax in high-risk patients is the best decision. However, the risk factors associated with pneumothorax that have been reported yet are controversial.

BMI is a commonly used indicator for assessing an individual’s obesity or wasting (29, 30). A cohort study found that patients in the lower BMI group had a higher risk of pneumothorax than those in the elevated BMI group (31). This study suggested that there may be a relationship between spontaneous pneumothorax and lower BMI. In a comprehensive investigation aimed at identifying risk factors associated with pneumothorax in Kuwait, Adel et al. (3) reported that individuals with a low BMI and males were more susceptible to developing pneumothorax. This phenomenon may be attributed to the fact that individuals with a lean physique tend to have a thinner chest wall fat and muscle. Consequently, their chest wall may be less effective in counteracting the pressure in the lungs, particularly during activities such as weight-bearing, sneezing, or forceful coughing. As a result, due to a sudden surge in lung pressure and the limited amount of subcutaneous fat and muscle in the chest wall, which is insufficient to offset the increased pressure in the lungs, there is a risk of damaging the pleura, and causing a pneumothorax (32). However, these studies were limited by the shortcomings of observational studies and could not determine whether a causal relationship actually existed (33). Our findings appear to be different from those of previous studies, we did not find a causal relationship between BMI and pneumothorax or spontaneous pneumothorax using two-samples Mendelian randomization. Lower BMI was not associated with the risk of developing either pneumothorax or spontaneous pneumothorax. We postulate that the association between BMI and pneumothorax in prior observational studies could be confounded by various factors, including smoking history, gender, underlying conditions, and work environment. Consequently, the findings of the MR analysis are deemed to be more compelling.

Recently, an observational study showed that no independent relationship was found between pneumothorax and height or lung function, even in patients with COPD (34). In contrast, several studies have suggested that there may be a potential relationship between height and the risk of spontaneous pneumothorax (2, 35). Some researchers have also indicated that taller patients may have a higher chance of suffering from pneumothorax (36, 37). In our study, higher height was associated with a greater risk of pneumothorax (OR > 1, *p* < 0.05). The mechanism of how higher height leads to pneumothorax is currently unclear. This phenomenon may be explained from anatomical and physiological perspectives. Anatomically, this may be related to the characteristics of the growing chest structure. Specifically, this condition may arise from the substantial increase in height during puberty among taller patients, resulting in lung apex thinning and congenital hypoplasia of the visceral pleura at the lung apex, thereby facilitating the formation of pulmonary bullae on the lung surface. Under increased chest pressure, these pulmonary bullae rupture and give rise to spontaneous pneumothorax (2, 38). Moreover, tall people have a longer longitudinal axis of the chest, which leads to increased mechanical stress on the tips of the lungs, making them more prone to rupture (39, 40). Physiologically, tall people have differences in lung compliance and elastic retraction, which may cause alveoli to rupture more easily under pressure changes (24). In addition, increased airflow resistance and turbulence in tall people will lead to increased mechanical stress in lung tissue, while the pressure difference in the lung will also produce higher stress in the upper lobe, making it more prone to rupture (41). To better establish the causal association between height (or BMI) and pneumothorax, we made the following key recommendations for future epidemiological and pathophysiological studies: (1) Large-scale GWAS studies across multiple regions and ethnicities; (2) Promote multidisciplinary cooperation, combining various research methods such as epidemiology, pathophysiology, genomics and imaging to jointly study the relationship between BMI (or height) and pneumothorax.

There were two obvious advantages in our study. On the one hand, the MR method was based on data related to published GWAS and has a large sample size of genetic variation, which makes the results more convincing. Confounders and reverse causation, on the other hand, were largely controlled for in this study. Nevertheless, our research also had several limitations. First, SNPs might be over-identification or have only a modest effect on the exposure factor (height or BMI) in a two-sample MR analysis (42, 43). Our analysis might have only a limited ability to detect associations. Second, all of the original GWAS data related to pneumothorax currently included in the GWAS database are most finely categorized to spontaneous pneumothorax, and there are no data that accurately categorize primary and secondary spontaneous pneumothorax. Based on this limitation, we were unable to conduct further subgroup analyzes of the causal relationship between BMI or height and primary and secondary spontaneous pneumothorax. Although this is one of the limitations of this study, MR analysis is used to more accurately assess the causal relationship between exposure factors and disease by exploiting the randomness of genetic variation, and therefore do not affect the reliability and robustness of the overall results. Further research on the association of BMI or height with different types of spontaneous pneumothorax will be the next research focus of our work. Third, gender and age may have some influence on the risk of pneumothorax, but we excluded gender- and age-related SNPs during our rigorous screening of IVs, so our findings were not affected by these confounders. Furthermore, Studies of height or BMI and pneumothorax were based on participants of European descent. There are differences in genetic susceptibility, smoking habits, living environment, and occupational exposures among people of different origins and regions, all of which may contribute to variations in the incidence of pneumothorax. Therefore, larger, multiregional, and multinational GWAS studies are needed to further characterize the association between height or BMI and pneumothorax.

## Conclusion

Our findings indicated that there was a significant causal relationship between height and pneumothorax, with height increasing the risk of developing pneumothorax. However, there was no causal relationship between BMI and pneumothorax and that reversed the understanding of previous observational studies. The relationship between BMI and pneumothorax requires further detailed analysis. Our findings provided a reference for decision making in clinical management and prevention of patients at risk of pneumothorax in the clinic. Although our findings were different from previous clinical experience and observational studies, further randomized controlled studies and basic experiments are needed to validate it, and ultimately to explore the deeper mechanisms by which height influences the development of pneumothorax and to produce more robust results.

## Data availability statement

The datasets presented in this study can be found in online repositories. The names of the repository/repositories and accession number(s) can be found in the article/Supplementary material.

## Ethics statement

Ethical approval was not required for the study involving humans in accordance with the local legislation and institutional requirements. Written informed consent to participate in this study was not required from the participants or the participants' legal guardians/next of kin in accordance with the national legislation and the institutional requirements.

## Author contributions

GL: Data curation, Formal analysis, Methodology, Software, Validation, Writing – original draft, Writing – review & editing, Resources, Visualization. DY: Data curation, Funding acquisition, Project administration, Visualization, Software, Writing – original draft. XW: Funding acquisition, Investigation, Resources, Writing – original draft. AL: Conceptualization, Data curation, Investigation, Validation, Writing – original draft, Writing – review & editing. JZ: Project administration, Supervision, Writing – review & editing.

## Footnotes

^1^ http://www.phenoscanner.medschl.cam.ac.uk/

## Supplementary material

Supplementary Figure S1 Funnel plot of the four independent MR analyzes. **(A)** Funnel plot of BMI genetic liability effects on pneumothorax. **(B)** Funnel plot of height genetic liability effects on pneumothorax. **(C)** Funnel plot of BMI genetic liability effects on spontaneous pneumothorax. **(D)** Funnel plot of height genetic liability effects on spontaneous pneumothorax.
