# Supplementary figures and images for: Novel insights into causal associations of body mass index or height with pneumothorax: a two-sample Mendelian randomization study
Source: Front Nutr. 2024 Jul 22;11:1391017. doi: 10.3389/fnut.2024.1391017 (PMC11298425; doi:10.3389/fnut.2024.1391017)

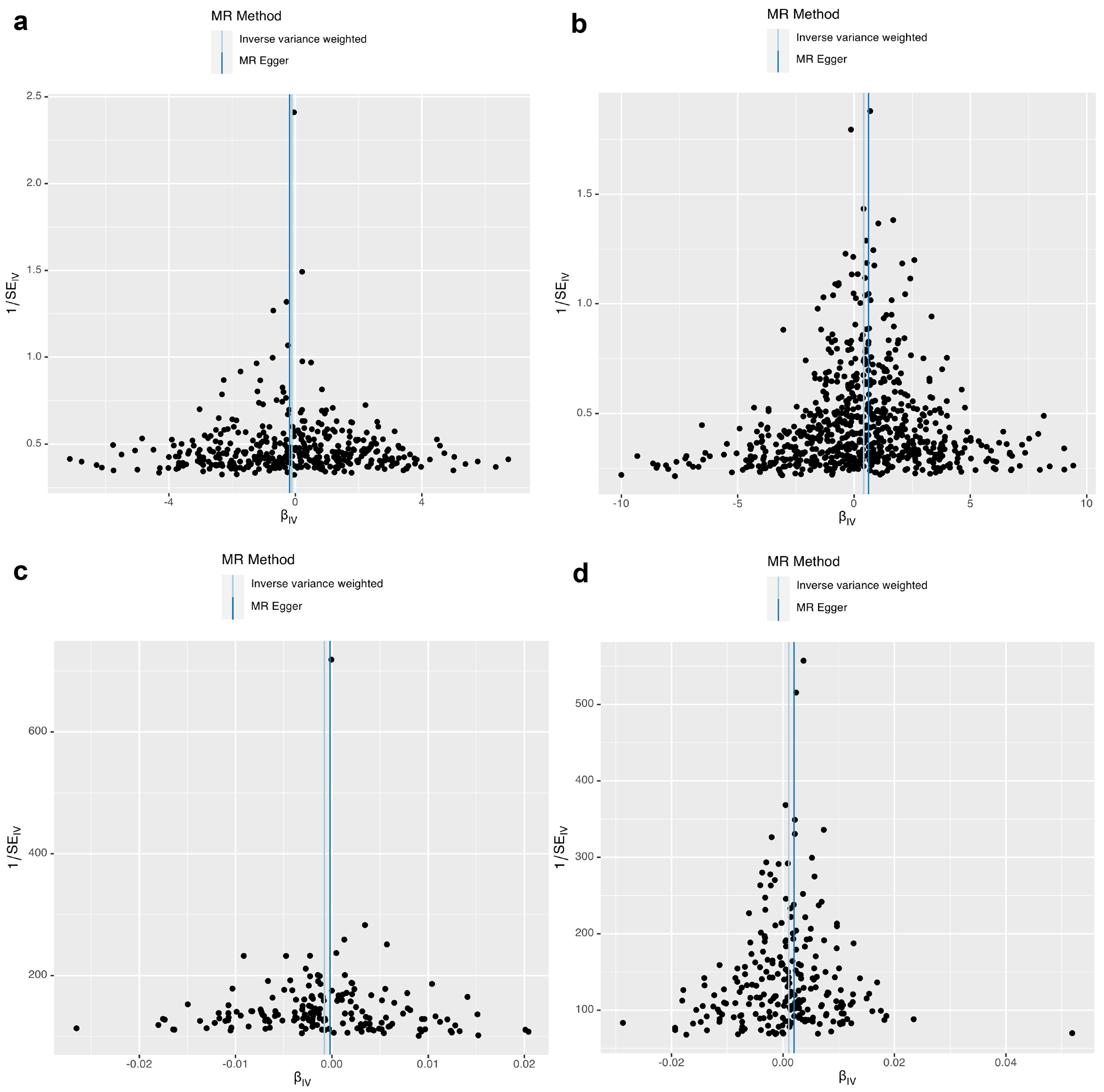

Supplement: Supplementary Figure S1 — Funnel plot of the four independent MR analyzes. (A) Funnel plot of BMI genetic liability effects on pneumothorax. (B) Funnel plot of height genetic liability effects on pneumothorax. (C) Funnel plot of BMI genetic liability effects on spontaneous pneumothorax. (D) Funnel plot of height genetic liability effects on spontaneous pneumothorax. [file Image_1.TIF]
